# Asymptomatic Hyperamylasemia in Stevens-Johnson Syndrome Is Associated with Intestinal Barrier Dysfunction

**DOI:** 10.1155/2020/3531907

**Published:** 2020-12-17

**Authors:** Yujen Tseng, Zhongguang Luo, Hongyang Zhang, Chengfeng Zhang, Jian Chen

**Affiliations:** ^1^Department of Digestive Diseases, Huashan Hospital, Fudan University, China; ^2^Department of Dermatology, Huashan Hospital, Fudan University, China

## Abstract

**Methods:**

A retrospective study on SJS patients was conducted at a tertiary medical center. All patients diagnosed as SJS, with available serum amylase index, were included. Clinical data of all subjects were retrospectively collected and analyzed. Colonic mucosal biopsies were obtained to measure tight junction protein expression.

**Results:**

A total of nine patients were included in the present study for study analysis. The average serum amylase of the study cohort was 228.78 ± 204.18 U/L. Among which, five patients had a positive fecal occult blood test (FOBT). Colonic mucosal biopsies were obtained and stained with occludin and zonula occludens-1 (ZO-1). The expression of occludin and ZO-1 was significantly downregulated in SJS patients (*p* < 0.01), which was indicative of intestinal barrier dysfunction.

**Conclusion:**

Hyperamylasemia often extends beyond pancreatic diseases. Clinical awareness of asymptomatic hyperamylasemia secondary to other systemic diseases can help avoid unnecessary overexamination and overtreatment.

## 1. Introduction

Hyperamylasemia is often regarded as the hallmark of acute pancreatitis in clinical practice. Apart from elevated pancreatic enzymes, acute pancreatitis is associated with concurrent signs and symptoms, such as abdominal pain, nausea, vomiting, hyperglycemia, or hypocalcemia [1]. Imaging studies may also reveal parenchymal enlargement and exudates, which further supports the diagnosis. However, asymptomatic hyperamylasemia is a rare presentation of acute pancreatitis and is more often associated with other systemic diseases such as mumps, tumors, kidney failure, or use of certain medications [2]. Poor recognition of asymptomatic hyperamylasemia in clinical practice may lead to unnecessary diagnostics test, inappropriate medical treatment, and waste of medical resources.

Stevens-Johnson syndrome (SJS) is a rare but serious and life-threatening disease of the skin and mucosa. SJS is characterized by the peeling of the skin, with a different degree of skin involvement, accompanied with blisters, sores, fever, and body aches. Extensive skin involvement of >30% is known as toxic epidermal necrolysis (TEN) [3]. More than half of the cases are associated with an allergic reaction to drugs, such as antibiotics or anticonvulsants. Bacterial infection, vaccination, or graft-versus-host diseases are also known to cause SJS and TEN. Patients are often admitted to the burn unit or intensive care unit and require administration of fluids and electrolytes. Corticosteroids and immunoglobulin have been proven to be beneficial in some cases, while others reported success with plasma exchange [4]. However, the most important treatment is to discontinue any suspicious drugs that may have triggered the allergic cascade [5].

Due to the nature of the disease, SJS and TEN may involve the skin and mucosa, including the mouth, eyes, vagina, urethra, respiratory tract, and gastrointestinal tract. Chosidow et al. was the first to reported hyperamylasemia in TEN, and there were few similar reports thereafter; however, the exact mechanism remains to be elucidated. Studies have shown an increase in amylase and lipase in SJS and TEN patients, with a reported incidence of 32.8% to 40%, suggesting mucous membrane involvement. However, authors concluded that these clinical phenomena should not preclude enteral nutrition as traditional clinical practice may dictate [6–8]. The present study is aimed at investigating the incidence of asymptomatic hyperamylasemia in SJS and clarifies the underlying mechanisms.

## 2. Materials and Methods

A retrospective study was conducted on patients diagnosed with Stevens-Johnson syndrome (SJS) at a tertiary medical center from August 2005 through August 2018. All patients with a confirmed diagnosis of SJS and available serum amylase index were included. Clinical data of all subjects were retrospectively collected and analyzed.

Patients eligible for study inclusion underwent colonoscopy examination, and biopsy of colonic mucosa was obtained for immunohistochemistry staining. Routine upper abdominal CT was ordered for all patients with elevated serum amylase to rule out pancreatitis. The tight junction protein occludin and zonula occludens-1 (ZO-1) were measured in the tissue samples. All patients signed an informed consent, acknowledging the purpose and risk associated with endoscopic examination. The study protocol was approved by the institution's ethics committee.

## 3. Tissue Specimen and Immunohistochemistry (IHC) Analysis

Tissue samples of colonic mucosa were embedded in paraffin. IHC staining of occludin (Abcam, UK) and ZO-1 (Cell Signaling Technology, USA) was carried out according to manufacturer's protocol.

Image analysis of IHC staining was achieved with the ImageJ software (Wayne Rasband, NIH, USA). Three random microscopic fields were selected for each tissue sample. Microscopic images were captured under 200x magnification for analysis. All images were analyzed under “RGB stack” mode, and the “threshold” was manually adjusted for optimal contrast to highlight the positively stained area or region of interest (ROI). Subsequently, the minimum, maximum, and mean gray values as well as area fraction (%) were calculated for comparison (Table 1).

## 4. Statistical Analysis

All statistical analyses were performed with SPSS 22 (IBM Corporation, USA). Categorical variables were presented as frequency (%), while continuous variables as mean ± standard deviation. Comparison between continuous variables was compared using the independent Student's *t*-test. All statistical analyses were two-sided, and a *p* value < 0.05 was considered statistically significant.

## 5. Results

**Figure 1:**
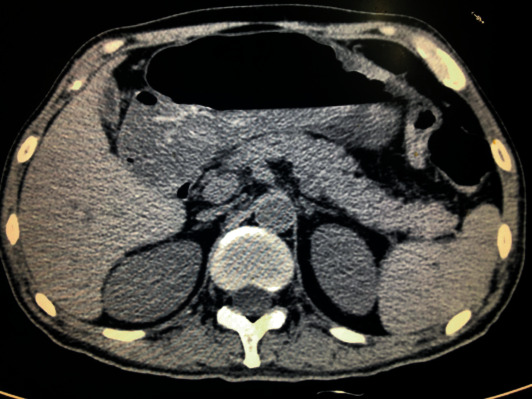
Stevens-Johnson syndrome. Routine upper abdominal CT to rule out acute pancreatitis of Patient 1, with a corresponding serum amylase of 663 U/L.

A total of 149 patients were diagnosed with SJS between August 2008 and August 2018 and were hospitalized at a tertiary medical center. Among which, 9 patients had available pancreatic enzymes for analysis and were included in the present study. The study subject included 6 (66.67%) male and 3 (33.33%) female patients, with an average age of 38.22 ± 16.20 years old. Clinically, patients who reported midabdominal pain or tenderness were tested for pancreatic enzymes to rule out acute pancreatitis. The average serum amylase was 228.78 ± 204.18 U/L, the average serum lipase was 270.07 ± 354.03 U/L (*n* = 7), the average blood glucose (BG) was 5.53 ± 1.05 mmol/L, and the average alanine amino*trans*ferase (ALT) and aspartate aminotransferase (AST) were 67.44 ± 82.01 U/L and 25.78 ± 14.49 U/L, respectively. Fecal occult blood test (FOBT) was positive among 5 (55.67%) patients, ranging from ± to +++, while 4 (44.44%) were negative. Subsequent abdominal CT revealed a nondiscrete pancreas without obvious swelling or exudates (Figure 1). Detailed patient characteristics are listed in Table 2. The serum indices of patients with positive FOBT were compared to patients with negative FOBT. Patients with positive FOBT had a significantly higher serum amylase and lipase, *p* < 0.05 (Table 3).

**Figure 2:**
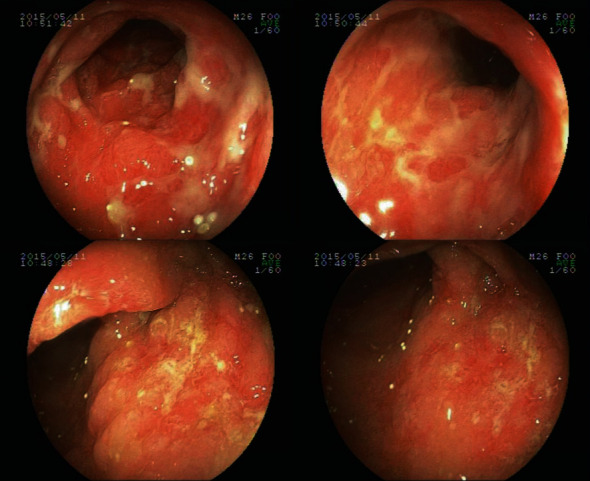
Stevens-Johnson syndrome. Diffuse mucosal inflammation with shallow ulcers localized to the sigmoid colon and rectum of Patient 1.

**Figure 3:**
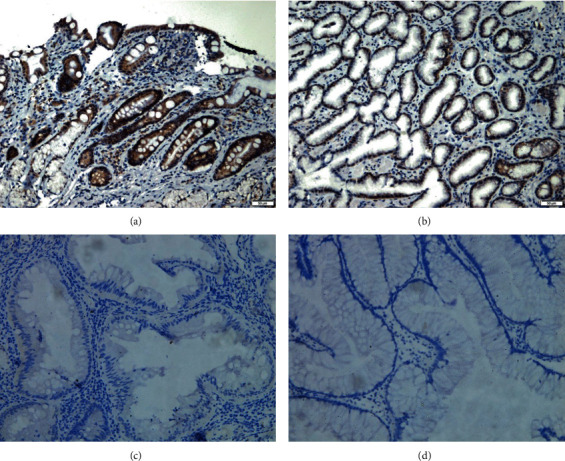
Stevens-Johnson syndrome. Immunohistochemistry staining of tight junction proteins. (a, c) show a significant downregulation of occludin (a) and ZO-1 (c) proteins in SJS patients, compared to healthy control (b, d).

Among patients with positive FOBT, 2 patients agreed to undergo colonoscopy examination and mucosal biopsy. Colonoscopy examination of one of the study subjects (Patient 1) revealed diffuse mucositis with shallow ulcers, mainly localized in the sigmoid colon and rectum (Figure 2). Pathology report revealed active mucosal inflammation with ulcer formation. The tight junction proteins, occludin and zonula occludens-1 (ZO-1), were measured by immunohistochemistry, while the colonic mucosa of a healthy volunteer served as control (Figure 3). No significant differences between mean gray values of patients and control were noted. Image analysis of three random visual fields of occludin and ZO-1 calculated a positive area fraction (%) of 5.83%, 8.77%, and 7.39% and 3.46%, 3.65%, and 3.64, respectively. The expression of protein occludin and ZO-1 was significantly downregulated in SJS patients (*p* < 0.01), which was indicative of intestinal barrier dysfunction (Table 1).

## 6. Discussion

Serum amylase, responsible for the breakdown of glucose and starch, can be categorized into salivary amylase (S-amylase) and pancreatic amylase (P-amylase). Generally, S-amylase and P-amylase account for 60% and 40% of total serum amylase activity, respectively. S-amylase is mainly secreted by the salivary glands but also by other organs, including the lungs, sweat glands, breast, gastrointestinal tract, and genitourinary system. Malignant tumors are also known to synthesize and secrete S-amylase. On the other hand, P-amylase is secreted by the pancreas and possesses a higher specificity for the diagnosis of pancreatic diseases, such as acute pancreatitis [9]. However, most clinical facilities fail to differentiate between S-amylase and P-amylase [10, 11]. Thus, clinical judgment of a physician is crucial in determining the significance of elevated serum amylase [12].

SJS is a rare allergic reaction with extensive involvement of the skin and mucosa. Among the present study cohort, the prevalence of hyperamylasemia is roughly 6%. Although patients reported suspicious abdominal symptoms with elevated serum amylase, the corresponding imaging examinations such as abdominal ultrasound or CT scan of the pancreas showed no obvious swelling or exudation. Therefore, the diagnosis of acute pancreatitis remains dubious. More than half the patients with SJS with accompanying hyperamylasemia had a positive FOBT. Subsequent colonoscopy examination and IHC results of the colonic mucosa revealed significant downregulation of tight junction protein (TJP), including occludin and ZO-1. Occludin is a protein encoded in the human *OCLN* gene, responsible for tight junction stability and barrier function [13]. Zonula occludens-1 (ZO-1), also known as tight junction protein-1, is a peripheral membrane protein encoded in the *TJP1* gene [14]. ZO-1 is involved in the signal transduction of cell-cell junction, which interacts with occludin to provide cytoplasmic membrane stability [14, 15].

Intestinal barrier injury secondary to SJS results in the reabsorption of amylase and lipase into the peripheral circulation, resulting in asymptomatic hyperamylasemia [16]. Based on this predisposition, SJS patients with asymptomatic hyperamylasemia should not only avoid fasting but also encourage enteral nutrition to promote recovery of intestinal mucosal barriers [17, 18]. Previous studies have shown that intestinal probiotics [19], glutamine, and Traditional Chinese Medicine (TCM) remedies, such as Sijunzi Tang and Kangfuxin Ye, have varying degrees of mucosal reinstitution and should be used flexibly to enhance healing of the intestinal mucosal barrier [20–22].

Boxberg et al. reported a 34-year-old woman admitted for treatment for toxic epidermolysis of the skin and mucosa. The patient died within 14 days due to persistent blood-streaked stools, bilateral pneumonia, and progressive respiratory failure. Further autopsy confirmed the cause of death as adult respiratory distress syndrome (ARDS) and complete loss of colonic mucosa [23].

The present study has several limitations, including the small sample size. Only nine patients had available enzymatic studies eligible for study inclusion; thus, the prevalence of hyperamylasemia in SJS may be understated. Correlation between prognosis and intestinal barrier dysfunction requires further examination. Currently, SCROTEN is a scoring system use to predict mortality rates of patients with SJS, a swell as burn victims. The scaling system includes age, associated malignancy, heart rate, serum BUN, detached or compromised body surface, serum bicarbonate, and serum glucose. Based on the present study cohort, positive FOBT indicative of mucosal barrier dysfunction can potentially be used as a prognostic indicator, warranting a larger study cohort with long-term follow-up.

The cause of hyperamylasemia is very complex, extending beyond pancreatic and salivary gland diseases. A small fraction of healthy population can also have a slight increase in serum amylase without clinical signs and symptoms. Therefore, when encountered with hyperamylasemia without clinical manifestations of pancreatitis and changes in pancreas morphology, subsequent amylase isoenzyme analysis should be done to rule out pancreatic diseases. For patients with SJS, asymptomatic hyperamylasemia secondary to intestinal mucosal injury should be recognized by dermatologists and gastroenterologists alike, to avoid unnecessary overexamination and overtreatment.

## Figures and Tables

**Table 1:** Image analysis of IHC result.

	Case	Control	*p* value
Occludin			
Mean gray value	170.11 ± 3.42	128.08 ± 30.34	0.076
Area fraction	3.58 ± 0.11	10.09 ± 0.92	<0.001
ZO-1			
Mean gray value	138.30 ± 30.02	129.53 ± 37.29	0.767
Area fraction	7.33 ± 1.47	15.14 ± 1.17	0.002

^∗^The area of all images was 13.65, captured under 200x magnification.

**Table 2:** General patient characteristics.

	Global population (*n* = 9)	Patient	Gender	Age	Amylase	Lipase	BG	ALT	AST	FOBT
Gender Male/female	6 (66.7%)/3 (33.3%)	1	M	20	663	555.5	7	82	50	+++
Age	38.22 ± 16.20	2	M	26	479	862.2	3.9	17	15	+
Amylase	228.78 ± 204.18	3	M	59	218	–	5.9	51	39	+
Lipase	239.7 ± 333.02	4	F	49	168	–	5.6	17	11	±
BG	5.53 ± 1.05	5	F	51	140	31	7.2	13	12	-
ALT	67.44 ± 82.01	6	M	24	132	56.6	4.8	270	36	-
AST	25.78 ± 14.49	7	M	28	103	57.5	4.8	101	34	-
FOBT Positive/negative	5 (55.6%)/4 (44.4%)	8	F	27	79	85.2	5.3	30	24	+
		9	M	60	77	29.9	5	26	11	-

**Table 3:** Comparison between patients with positive and negative FOBT.

	Positive FOBT (*n* = 5)	Negative FOBT (*n* = 4)	*p* value
Gender			
Male	3 (60.0%)	3 (75.0%)	0.635
Female	2 (40.0%)	1 (25.0%)	
Age	36.2 ± 16.84	40.75 ± 17.50	0.882
Amylase	321.40 ± 242.13	113.00 ± 28.79	0.008
Lipase	500.97 ± 391.36	43.75 ± 15.37	0.038
BG	5.48 ± 1.14	5.60 ± 1.09	0.957
ALT	39.40 ± 27.57	102.50 ± 118.21	0.082
AST	27.80 ± 16.42	23.25 ± 13.60	0.658

## Data Availability

The data used in the present study will be made available upon request to the corresponding author.
